# Identification and Molecular Characterization of a Novel *Carlavirus* Infecting *Chrysanthemum morifolium* in China

**DOI:** 10.3390/v15041029

**Published:** 2023-04-21

**Authors:** Jiapeng Li, Xiaoyin Wu, Hui Liu, Xiaomei Wang, Shaokui Yi, Xueting Zhong, Yaqin Wang, Zhanqi Wang

**Affiliations:** 1 Key Laboratory of Vector Biology and Pathogen Control of Zhejiang Province, College of Life Sciences, Huzhou University, Huzhou 313000, China; ljpbangbangde@163.com (J.L.); wxy2452675088@163.com (X.W.); 01960@zjhu.edu.cn (X.W.); yishaokui@foxmail.com (S.Y.); zxt@zjhu.edu.cn (X.Z.); 2 State Key Laboratory of Rice Biology, Institute of Biotechnology, Zhejiang University, Hangzhou 310058, China; 12216096@zju.edu.cn

**Keywords:** *Chrysanthemum morifolium*, *Carlavirus*, phylogenetic analysis, host jump, pathogenesis

## Abstract

Chrysanthemum (*Chrysanthemum morifolium*) is an important ornamental and medicinal plant suffering from many viruses and viroids worldwide. In this study, a new carlavirus, tentatively named Chinese isolate of *Carya illinoinensis* carlavirus 1 (CiCV1-CN), was identified from chrysanthemum plants in Zhejiang Province, China. The genome sequence of CiCV1-CN was 8795 nucleotides (nt) in length, with a 68-nt 5′-untranslated region (UTR) and a 76-nt 3′-UTR, which contained six predicted open reading frames (ORFs) that encode six corresponding proteins of various sizes. Phylogenetic analyses based on full-length genome and coat protein sequences revealed that CiCV1-CN is in an evolutionary branch with chrysanthemum virus R (CVR) in the *Carlavirus* genus. Pairwise sequence identity analysis showed that, except for CiCV1, CiCV1-CN has the highest whole-genome sequence identity of 71.3% to CVR-X6. At the amino acid level, the highest identities of predicted proteins encoded by the ORF1, ORF2, ORF3, ORF4, ORF5, and ORF6 of CiCV1-CN were 77.1% in the CVR-X21 ORF1, 80.3% in the CVR-X13 ORF2, 74.8% in the CVR-X21 ORF3, 60.9% in the CVR-BJ ORF4, 90.2% in the CVR-X6 and CVR-TX ORF5s, and 79.4% in the CVR-X21 ORF6. Furthermore, we also found a transient expression of the cysteine-rich protein (CRP) encoded by the ORF6 of CiCV1-CN in *Nicotiana benthamiana* plants using a potato virus X-based vector, which can result in a downward leaf curl and hypersensitive cell death over the time course. These results demonstrated that CiCV1-CN is a pathogenic virus and *C. morifolium* is a natural host of CiCV1.

## 1. Introduction

Chrysanthemum (*Chrysanthemum morifolium*) is a herbaceous perennial plant in the family *Asteraceae*, and has important ornamental, economic, and medicinal value [1,2,3]. Chrysanthemum flowers have been made into traditional herbal remedies in China due to their potential effects on treating respiratory and cardiovascular diseases [4,5,6]. Commercially, the chrysanthemum is propagated mainly through the stem or root cuttings [7,8]. However, these reproduction methods frequently lead to the accumulation and spread of harmful pathogens [8,9], which finally cause the degradation of the chrysanthemum variety and reductions in flower yield and quality. In recent years, with the rapid expansion of chrysanthemum-growing areas, diseases caused by pathogens have become one of the most severe problems in chrysanthemum production worldwide [10,11,12,13,14].

In China, disease symptoms, such as chlorosis, mosaic, mottling, and stunting, are frequently detected in chrysanthemum plants. In 2019, the disease incidence of chrysanthemum was estimated at approximately 100% in Zhejiang Province, China, the main *C. morifolium* production region [15,16]. Astonishingly, viral and viroid infections cause up to 30% of the losses of infected chrysanthemum plants [10,17]. So far, more than 20 viruses and viroids have been identified to infect chrysanthemum [6,13,16], the most prevalent of which are typically considered to be chrysanthemum virus B (CVB) [16,18], chrysanthemum virus R (CVR) [19], cucumber mosaic virus (CMV) [10], tomato aspermy virus (TAV) [20], tobacco mosaic virus (TMV) [10], tomato spotted wilt virus (TSWV) [21], potato virus Y (PVY) [22], chrysanthemum stunt viroid (CSVd) [18], and chrysanthemum chlorotic mottle viroid (CChMVd) [23]. It is important to note that the main prevalent CVB and CVR both belong to the *Carlavirus* genus in the *Betaflexiviridae* family [13,18,19]. Therefore, the characterization of new species of *Carlavirus* from chrysanthemum is a critical aspect of our understanding of the molecular mechanisms underlying the response of plants to viral infections.

The *Carlavirus* genus is a diverse group of plant viruses with a positive-sense, single-stranded RNA genome and a filamentous virion [6,13,24,25,26]. Carlaviruses are able to infect many plants, including some important economic and horticultural crops, resulting in substantial yield and financial losses worldwide [25,26,27,28,29]. Carlaviruses can be transmitted from plant to plant by asexual reproduction and/or aphids in a non-persistent manner [6,13,24]. The genomes of carlaviruses are approximately 8.0–9.0 kilobases in length, with a 5′-cap structure and a 3′-poly(A) tail, which encompass six open reading frames (ORFs) [6,13,19,24,26]. ORF1 encodes a replicase-related protein with main domains for methyltransferase, endopeptidase, RNA helicase, and RNA-dependent RNA polymerase (RdRP); ORFs 2–4 encode three triple gene block (TGB) proteins (TGBp1, TGBp2, and TGBp3) that are involved in viral movement and host cell membrane modification; ORF5 encodes a coat protein (CP), and ORF6 encodes a cysteine-rich protein (CRP) that has a conserved nuclear localization signal (NLS) and an adjacent zinc finger (ZF) motifs, which may be related to the pathogenicity of *Carlavirus* [13,19,30].

To date, two major species, *Carlavirus* CVB and CVR, have been characterized with full-length genomes from chrysanthemum plants. For *Carlavirus* CVB, the complete genome sequence of Japanese isolate (CVB-S) was the first to be obtained in 2007 [24]. In 2012, four complete genome sequences of CVB-TN, CVB-PB, CVB-UP, and CVB-UK isolates from India were released [31]. More recently, the complete genome sequences of three Russian isolates (CVB-GS1, CVB-GS2, and CVB-FY) and two Chinese isolates (CVB-CN2 and CVB-CN5) were obtained using next-generation sequencing (NGS) [6,13]. For *Carlavirus* CVR, the first one with a complete genome was the Chinese isolate (CVR-BJ) obtained in 2018 [19]. Subsequently, whole-genome sequences of three Russian isolates (CVR-6, CVR-13, and CVR-21) and another three Chinese isolates (CVR-TX, CVR-ZJHU1, and CVR-ZJHU2) were obtained from chrysanthemum plants in Zhejiang Province, China [13,32,33]. These genome sequences of *Carlavirus* from the chrysanthemum provide valuable materials for studying the pathogenicity mechanisms and functional genes of carlaviruses.

In this study, we identified a new carlavirus (tentatively named *Carya illinoinensis* carlavirus 1 Chinese isolate, CiCV1-CN) from a chrysanthemum sample collected from Zhejiang Province, China. Subsequently, we analyzed the genomic organization and phylogeny of this virus. We also determined the pathogenicity of the CRP of CiCV1-CN (CiCV1-CN CRP) using a potato virus X (PVX)-based vector. Our results revealed that CiCV-CN is a new member of the *Carlavirus* genus. This is the first report that characterizes CiCV1 infecting chrysanthemum plants naturally. These findings provide strong evidence of the cross-species transmission of CiCV1 and improve our understanding of the molecular mechanisms of the transmission and pathogenesis of carlaviruses.

## 2. Materials and Methods

### 2.1. Plant Sample Collection

**Figure 1:**
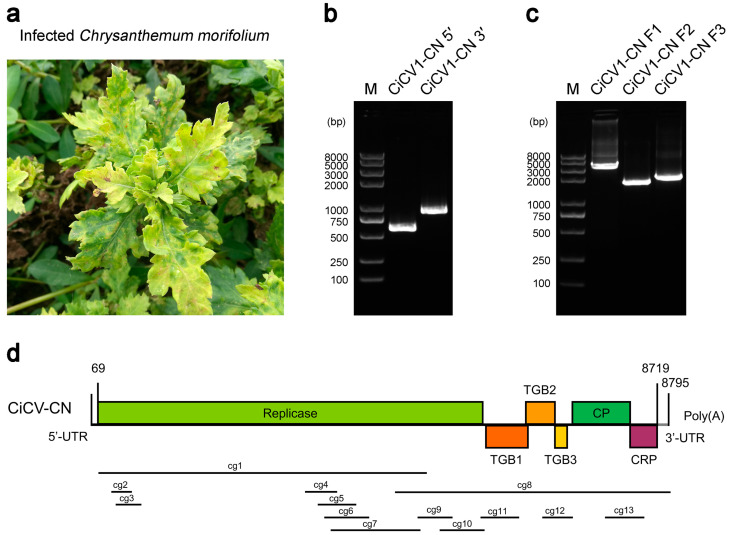
Symptoms, molecular cloning, and genome structure of the Chinese isolate of *Carya illinoinensis* carlavirus 1 (CiCV1-CN) from *Chrysanthemum morifolium*. (**a**) Symptoms of CiCV1-CN in *C. morifolium*. (**b**) 5′- and 3′-RACE (rapid amplification of cDNA ends) cloning of CiCV1-CN. M: DNA marker. (**c**) Reverse transcription PCR (RT-PCR) cloning of internal genomic fragments (F1, F2, and F3) of CiCV1-CN. M: DNA marker. (**d**) Genomic organization of CiCV1-CN. The predicted open reading frames (ORFs) are marked with different rectangles, and 13 contigs assembled from virus-derived small RNAs are indicated with black lines.

In October 2020, chrysanthemum (*C. morifolium*) leaf samples with chlorosis, vein yellowing, and clearing symptoms were collected from Zhejiang Province, China (Figure 1a). Leaf samples used for RNA extraction were immediately frozen in liquid nitrogen and stored at −80 °C until use.

### 2.2. NGS and Sequence Assembly

Total RNA was extracted from infected leaf samples using a TRIzol reagent (Invitrogen, Carlsbad, CA, USA) according to the manufacturer’s instructions. The RNA purity and quantity were determined using the Agilent 2100 Bioanalyzer (Agilent Technologies, Santa Clara, CA, USA) and Nanodrop Spectrophotometer (Thermo Scientific, Waltham, MA, USA). The ribosomal RNA was removed by the Epicentre Ribo-Zero^TM^ rRNA Removal Kit (Epicentre, Madison, WI, USA), and NGS was performed on the Illumina NovaSeq 6000 platform (Illumina, San Diego, CA, USA) with a paired-end 150 bp set-up, as described previously [6,34,35]. After removing the adapter and low-quality sequences, the resulting clean reads were assembled using Trinity (v.2.14.0) [36]. The assembled contigs were subsequently searched against the NCBI viral (NCBI txid: 10239) sequence database (https://www.ncbi.nlm.nih.gov/genome/viruses/, accessed on 12 April 2022), as described previously [34,37,38,39].

### 2.3. Amplification of the Full-Length Genome Sequence of CiCV1-CN

To confirm the NGS result and to understand the differences between CiCV1 isolates from different host plants, we cloned and sequenced the complete nucleotide sequence of CiCV1-CN isolated in *C. morifolium* from Zhejiang Province, China. To obtain the full-length genome sequence of CiCV1-CN, a rapid amplification of cDNA ends (RACE) technique was utilized [6,40]. We synthesized 5′- and 3′-RACE cDNAs using a SMARTer^®^ RACE 5′/3′ Kit (Takara Bio Inc., Dalian, China), following the manufacturer′s protocol. PCR amplification reactions were performed on a T100 PCR cycler (Bio-Rad, Pleasanton, CA, USA) using the KOD-plus DNA polymerase (Toyobo, Osaka, Japan). The primers used for the genome sequence cloning are listed in Supplementary Table S1. The obtained full-length genome sequence of CiCV1-CN was submitted to the NCBI Genbank database (https://www.ncbi.nlm.nih.gov/genbank/, accessed on 7 February 2023) under an accession number OQ410649.

### 2.4. Nucleic Acid and Protein Sequence Alignments

All viral genome sequences were downloaded from the NCBI nucleotide database (https://www.ncbi.nlm.nih.gov/nuccore/, accessed on 12 April 2022). The viral protein sequences were obtained from the NCBI protein sequence database (https://www.ncbi.nlm.nih.gov/protein/, accessed on 12 April 2022). Global nucleotide and amino acid sequence identities were calculated using the EMBOSS Needle (https://www.ebi.ac.uk/Tools/psa/emboss_needle/, accessed on 30 August 2022) [41]. A multiple sequence alignment of the replicase-related proteins, CPs, and CRPs was performed using the ClustalW program embedded in the Molecular Evolutionary Genetics Analysis software (MEGA, v11.0) [42]. Conserved domains of CPs and CRPs were analyzed using the NCBI Conserved Domain Database (CDD) (https://www.ncbi.nlm.nih.gov/cdd/, accessed on 30 August 2022) [43] and the InterPro (https://www.ebi.ac.uk/interpro/, accessed on 30 August 2022) [44].

### 2.5. Phylogenetic and Recombination Analyses

Multiple sequence alignment analyses of the genomes or proteins of CiCV1-CN and their closely related viruses were performed using the ClustalW program in the MEGA11 [42]. Phylogenetic trees were constructed using the MEGA11 [42] by the maximum-likelihood (ML) method with a GTR + G+I for genomes or by the neighbor-joining (NJ) method with a Jones–Taylor–Thornton (JTT) model for proteins, as described previously [6,38]. Recombination events were detected using the recombination detection program RDP4 (v4.101) [45], as described previously [13,33].

### 2.6. Plant Material and Growth Conditions

Wild-type *Nicotiana benthamiana* plants were used in this study, and they were grown in soil:vermiculite:perlite (3:3:1, *v/v/v*) at 25 ± 1 °C with a 16-h/8 h (light/dark) photoperiod, as described previously [46]. After 30 days of cultivation, the plants at the 5-leaf stage were used for viral inoculation.

### 2.7. PVX Construct and Viral Inoculation

To construct the PVX-based expression plasmids, the coding sequences of CRPs of CiCV1 and CiCV1-CN were cloned to PVX vector to generate PVX:CiCV1 CRP and PVX:CiCV1-CN CRP, respectively. After confirmation by sequencing, the constructed plasmids and empty vector were individually transferred into the *Agrobacterium tumefaciens* strain GV3101 by electroporation, as described previously [47,48]. The *Agrobacterium*-mediated inoculation of *N. benthamiana* was performed as described previously [49,50]. Simultaneously, the PVX-based vector expressing a green fluorescent protein (GFP) (PVX:GFP) was used as a vector control.

### 2.8. RNA Extraction and Quantitative PCR (qPCR) Analysis

The inoculated *N. benthamiana* plants were photographed at 0, 7, and 14 days post-inoculation (dpi); meanwhile, systemically infected leaves were sampled and frozen in liquid nitrogen. Total RNA extraction and cDNA synthesis were performed, as described previously [6]. qPCR was carried out on a CFX96 Touch Deep Well Real-Time PCR Detection System (Bio-Rad, Pleasanton, CA, USA), as described by Wang et al. [51]. *N. benthamiana actin 2* (*NbACT2*) was used as an internal reference [46,52]. The relative viral RNA accumulation levels were calculated by the comparative *C*_T_ method [53]. The reactions were performed in triplicate, and the results were averaged. The primers used for qPCR are listed in Supplementary Table S1.

### 2.9. Statistical Analysis

The data are presented as means ± standard deviation (SD) of three independent biological replicates. The statistical significance of differences was calculated using a Student′s *t*-test in Microsoft Excel (v. 2021, Microsoft Corp., Redmond, WA, USA). A *p*-value of less than 0.05 (*p* < 0.05) was considered statistically significant.

## 3. Results

### 3.1. NGS and Genomic Organization of CiCV1-CN

In total, 84,378,724 raw reads were obtained, and after removing the adaptor and low-quality sequences, 82,665,768 clean reads (12.4 G) were produced (Supplementary Table S2). As a result, 245,035 contigs were generated after de novo assembling using Trinity (v.2.14.0) [36]. Among them, 13 contigs with an alignment length of more than 300 nucleotides (nt) were found to have high alignment scores (E-value ˂ 1 × 10^–100^) with CiCV1 (GenBank: MW328759) that was deposited in the NCBI nucleotide database (https://www.ncbi.nlm.nih.gov/nuccore/, accessed on 7 February 2023) (Supplementary Table S3).

To obtain the full-length genome sequence of CiCV1-CN, 5′/3′ RACE technique and three-segment amplification strategy were used, and the sizes for the 5′ and 3′ ends and the three internal genomic fragments were 565, 990, 3753, 1861, and 2115, respectively (Figure 1b,c). The genome sequence of CiCV1-CN was 8795 nt in length (excluding poly(A) tail), and the sizes of the 5′-untranslated region (UTR) (5′-UTR) and 3′-UTR were 68 nt and 76 nt, respectively (Figure 1d). The genome of CiCV1-CN contained six predicted ORFs, which encoded six corresponding proteins of various sizes (Figure 1d). ORF1 (69–6221 nt) encodes a 232.3-kDa replicase-related protein that contains five important conserved domains: viral methyltransferase (IPR002588), oxoglutarate/iron-dependent dioxygenase (IPR005123), peptidase C23 (IPR008041), RNA virus helicase (IPR027351), and RdRP (IPR001788). ORF2 (6249–6938 nt), ORF3 (6916–7239 nt), and ORF4 (7239–7430 nt) encode three viral movement- and host cell membrane modification-related proteins (TGBp1, TGBp2, and TGBp3). ORF5 (7473–8396 nt) encodes a 34.4-kDa viral CP. ORF6 (8396–8719 nt) encodes an 11.9-kDa CRP. The complete genome sequence of CiCV1-CN was deposited in the NCBI GenBank (https://www.ncbi.nlm.nih.gov/genbank/, accessed on 7 February 2023) under an accession number OQ410649.

### 3.2. Phylogenetic and Recombination Analyses of CiCV1-CN

**Figure 2:**
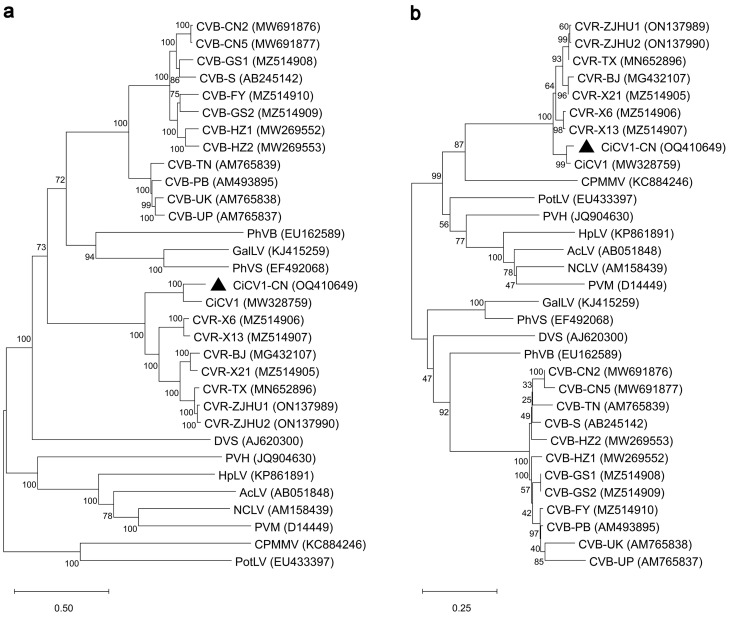
Phylogenetic relationships of the Chinese isolate of *Carya illinoinensis* carlavirus 1 (CiCV1-CN) and 31 reported carlaviruses. Phylogenetic trees were generated based on the full-length genome sequences (**a**) and coat protein sequences (**b**) using the Molecular Evolutionary Genetics Analysis software (MEGA, v11.0) with bootstrap values of 1000 replicates. The following viruses were used in the phylogenetic tree construction: aconitum latent virus (AcLV, AB051848), *Carya illinoinensis* carlavirus 1 (CiCV1, MW328759), *Carya illinoinensis* carlavirus 1 Chinese isolate (CiCV1-CN, OQ410649), chrysanthemum virus B isolate CN2 (CVB-CN2, MW691876), chrysanthemum virus B isolate CN5 (CVB-CN5, MW691877), chrysanthemum virus B isolate FY (CVB-FY, MZ514910), chrysanthemum virus B isolate GS1 (CVB-GS1, MZ514908), chrysanthemum virus B isolate GS2 (CVB-GS2, MZ514909), chrysanthemum virus B isolate HZ-V1 (CVB-HZ1, MW269552), chrysanthemum virus B isolate HZ-V2 (CVB-HZ2, MW269553), chrysanthemum virus B isolate Punjab (CVB-PB, AM493895), chrysanthemum virus B isolate S (CVB-S, AB245142), chrysanthemum virus B isolate Tamil Nadu (CVB-TN, AM765839), chrysanthemum virus B isolate Uttar Pradesh (CVB-UP, AM765837), chrysanthemum virus B isolate Uttarakhand (CVB-UK, AM765838), chrysanthemum virus R isolate TX (CVR-TX, MN652896), chrysanthemum virus R isolate X13 (CVR-X13, MZ514907), chrysanthemum virus R isolate X21 (CVR-X21, MZ514905), chrysanthemum virus R isolate X6 (CVR-X6, MZ514906), chrysanthemum virus R isolate ZJHU1 (CVR-ZJHU1, ON137989), chrysanthemum virus R isolate ZJHU2 (CVR-ZJHU2, ON137990), chrysanthemum virus R isolate BJ (CVR-BJ, MG432107), cowpea mild mottle virus (CPMMV, KC884246), daphne virus S (DVS, AJ620300), gaillardia latent virus (GalLV, KJ415259), hop latent virus (HpLV, KP861891), *Narcissus common* latent virus (NCLV, AM158439), phlox virus B (PhVB, EU162589), phlox virus S (PhVS, EF492068), potato latent virus (PotLV, EU433397), potato virus H (PVH, JQ904630), and potato virus M (PVM, D14449).

To further investigate the evolutionary relationship between CiCV1-CN and other carlaviruses, we constructed phylogenetic trees at the genome and protein levels. The phylogenetic analysis of the full-length genomes of CiCV1-CN and the 31 reported carlaviruses indicated that CiCV1-CN and CiCV-1 clustered together and formed a smaller branch adjacent to the CVR subcluster (Figure 2a). Pairwise sequence identity analysis showed that, except for CiCV1, CiCV1-CN has the highest whole-genome sequence identity of 71.3% to CVR-X6 and the lowest identity of 53.9% to cowpea mild mottle virus (CPMMV) (Supplementary Table S4). These sequence identities met the current species demarcation criteria for the *Carlavirus* genus [27,54,55]. These findings, therefore, suggest that CiCV1-CN is a new species of *Carlavirus*, which possesses a closer evolutionary relationship with *Carlavirus* CVR. Furthermore, the phylogenetic tree based on the CP sequences showed a similar clustering result (Figure 2b). Further amino acid sequence alignment demonstrated that CiCV1-CN CP displays high-sequence identities to CVR CPs (88.9–96.4%) (Figure S1a). Conserved domain analysis based on the InterPro (http://www.ebi.ac.uk/interpro/, accessed on 30 August 2022) [44] indicated that CiCV1-CN CP has a Carlavirus_coat_N domain (IPR013569, 62–112 aa) and a Pltvir_coat domain (IPR000052, 121–261 aa) in its N and C termini, respectively (Figure S1b), suggesting that CiCV1-CN CP possesses the typical properties of CPs of carlaviruses. Together, these results indicate that CiCV1-CN is a new virus species of *Carlavirus* from *C. morifolium*.

Previously, studies have shown that RNA recombinations are frequently observed in CVB and CVR viruses [13,31,33]. Therefore, we determined whether the CiCV1-CN has RNA recombinations with other carlaviruses. However, no recombination event was detected in the genome of CiCV1-CN (Supplementary Table S5).

### 3.3. Sequence Identity Analysis of Carlaviruses from C. morifolium

To further examine the sequence identities of CiCV1-CN with the other carlaviruses from *C. morifolium*, we analyzed the nucleotide and amino acid sequences of CiCV1-CN and the 17 reported carlaviruses identified from *C. morifolium*. The results revealed that CiCV1-CN had sequence identities from 71.3% to 56.2% in the other carlaviruses identified from *C. morifolium* at the whole-genome level (Table 1). Interestingly, the 5′-UTR CiCV1-CN showed the highest identity of 84.5% in the 5′-UTRs of CVR-ZJHU1, CVR-ZJHU2, and CVR-TX, while the 3′-UTR displayed the highest identity of 88.5% only in the 3′-UTR of CVR-TX (Table 1). At the amino acid level, the highest identities of predicted proteins encoded by the ORF1, ORF2, ORF3, ORF4, ORF5, and ORF6 were 77.1% in the CVR-X21 ORF1, 80.3% in the CVR-X13 ORF2, 74.8% in the CVR-X21 ORF3, 60.9% in the CVR-BJ ORF4, 90.2% in the CVR-X6 and CVR-TX ORF5s, and 79.4% in the CVR-X21 ORF6, respectively (Table 1).

**Figure 3:**
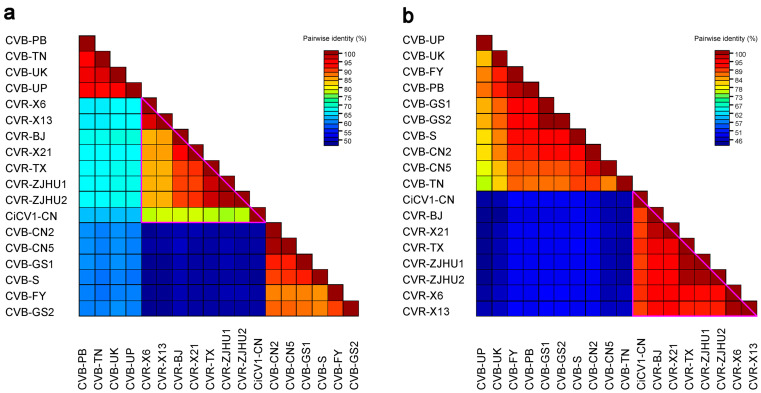
Heat map analysis of the pairwise identity matrixes of the Chinese isolate of *Carya illinoinensis* carlavirus 1 (CiCV1-CN) and 17 reported carlaviruses identified from *Chrysanthemum morifolium* at the whole replicase-related protein level (**a**) and the coat protein (CP) level (**b**). The purple triangle represents the high pairwise identity matrixes of CiCV1-CN to the *Carlavirus* chrysanthemum virus R (CVR). The following viruses were used in the heat map analysis: *Carya illinoinensis* carlavirus 1 Chinese isolate (CiCV1-CN, OQ410649), chrysanthemum virus B isolate CN2 (CVB-CN2, MW691876), chrysanthemum virus B isolate CN5 (CVB-CN5, MW691877), chrysanthemum virus B isolate FY (CVB-FY, MZ514910), chrysanthemum virus B isolate GS1 (CVB-GS1, MZ514908), chrysanthemum virus B isolate GS2 (CVB-GS2, MZ514909), chrysanthemum virus B isolate Punjab (CVB-PB, AM493895), chrysanthemum virus B isolate S (CVB-S, AB245142), chrysanthemum virus B isolate Tamil Nadu (CVB-TN, AM765839), chrysanthemum virus B isolate Uttar Pradesh (CVB-UP, AM765837), chrysanthemum virus B isolate Uttarakhand (CVB-UK, AM765838), chrysanthemum virus R isolate TX (CVR-TX, MN652896), chrysanthemum virus R isolate X13 (CVR-X13, MZ514907), chrysanthemum virus R isolate X21 (CVR-X21, MZ514905), chrysanthemum virus R isolate X6 (CVR-X6, MZ514906), chrysanthemum virus R isolate ZJHU1 (CVR-ZJHU1, ON137989), chrysanthemum virus R isolate ZJHU2 (CVR-ZJHU2, ON137990), and chrysanthemum virus R isolate BJ (CVR-BJ, MG432107).

Next, we performed pairwise matrix comparisons of CiCV1-CN amino acid sequences of the whole replicase-related proteins and CPs among the homologs of carlaviruses identified from *C. morifolium* using heat maps. As shown in Figure 3, CiCV1-CN dramatically clustered together with high-sequence identities to *Carlavirus* CVR at both the replicase-related protein and the CP levels. These results further suggest that CiCV1-CN is closer to the *Carlavirus* CVR.

### 3.4. Phylogenetic and Sequence Analyses of CRP Proteins of Carlaviruses from C. morifolium

**Figure 4:**
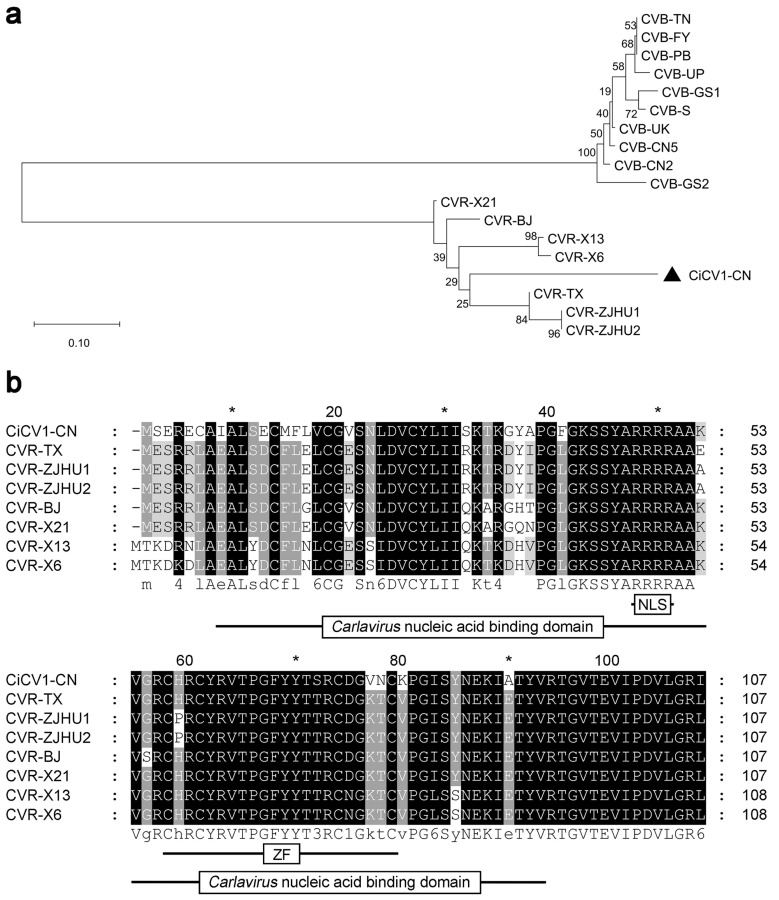
Phylogenetic and sequence analyses of cysteine-rich proteins (CRPs) encoded by carlaviruses from *Chrysanthemum morifolium*. (**a**) The phylogenetic tree was constructed based on the amino acid sequences of CRPs of carlaviruses from *C. morifolium* using the Molecular Evolutionary Genetics Analysis software (MEGA, v11.0) with bootstrap values of 1000 replicates. The following viruses were used in the phylogenetic tree construction: *Carya illinoinensis* carlavirus 1 Chinese isolate (CiCV1-CN, OQ410649), chrysan-themum virus B isolate CN2 (CVB-CN2, MW691876), chrysanthemum virus B isolate CN5 (CVB-CN5, MW691877), chrysanthemum virus B isolate FY (CVB-FY, MZ514910), chrysanthemum virus B isolate GS1 (CVB-GS1, MZ514908), chrysanthemum virus B isolate GS2 (CVB-GS2, MZ514909), chrysanthemum virus B isolate Punjab (CVB-PB, AM493895), chrysanthemum virus B isolate S (CVB-S, AB245142), chrysanthemum virus B isolate Tamil Nadu (CVB-TN, AM765839), chrysanthemum virus B isolate Uttar Pradesh (CVB-UP, AM765837), chrysanthemum virus B isolate Uttarakhand (CVB-UK, AM765838), chrysanthemum virus R isolate TX (CVR-TX, MN652896), chrysanthemum virus R isolate X13 (CVR-X13, MZ514907), chrysanthemum virus R isolate X21 (CVR-X21, MZ514905), chrysanthemum virus R isolate X6 (CVR-X6, MZ514906), chrysanthemum virus R isolate ZJHU1 (CVR-ZJHU1, ON137989), chrysanthemum virus R isolate ZJHU2 (CVR-ZJHU2, ON137990), and chrysanthemum virus R isolate BJ (CVR-BJ, MG432107). (**b**) Amino acid sequence alignment of CRPs of carlaviruses from *C. morifolium* using the ClustalW program embedded in Molecular Evolutionary Genetics Analysis software (MEGA, v11.0), and the conserved domains were determined using the InterPro (http://www.ebi.ac.uk/interpro/ (accessed on 30 August 2022)). The following viruses were used in the amino acid sequence alignments: *Carya illinoinensis* carlavirus 1 Chinese isolate (CiCV1-CN, OQ410649), chrysanthemum virus R isolate TX (CVR-TX, MN652896), chrysanthemum virus R isolate X13 (CVR-X13, MZ514907), chrysanthemum virus R isolate X21 (CVR-X21, MZ514905), chrysanthemum virus R isolate X6 (CVR-X6, MZ514906), chrysanthemum virus R isolate ZJHU1 (CVR-ZJHU1, ON137989), chrysanthemum virus R isolate ZJHU2 (CVR-ZJHU2, ON137990), and chrysanthemum virus R isolate BJ (CVR-BJ, MG432107). * indicates the position of odd tens of amino acids.

Previous studies have shown that the CRPs of the genus *Carlavirus* play a crucial role in causing viral symptoms [13,30,56]. We, therefore, examined the phylogenetic relationships of CiCV1-CN CRP with the other CRPs encoded by carlaviruses from *C. morifolium*. As expected, CiCV1-CN CRP was clustered closely with the CRPs encoded by the *Carlavirus* CVR (Figure 4a). This result suggests that the relationship between CiCV1-CN CRP and *Carlavirus* CVR CRPs is closer during the evolution. Further amino acid sequence alignment of CRPs showed that CiCV1-CN CRP had a conserved *Carlavirus* nucleic acid binding domain (IPR002568, 8–92 aa) (Figure 4b). Furthermore, CiCV1-CN CRP was also predicted to have an arginine-rich NLS motif (^47^RRRR^50^) and a ZF motif (^57^CX_2_CX_12_CX_4_C^78^) located adjacent to the NLS (Figure 4b). These results suggest that CiCV1-CN CRP may be a potential pathogenicity factor during viral infection.

### 3.5. CiCV1-CN CRP Is a Pathogenicity Factor of CiCV1-CN

**Figure 5:**
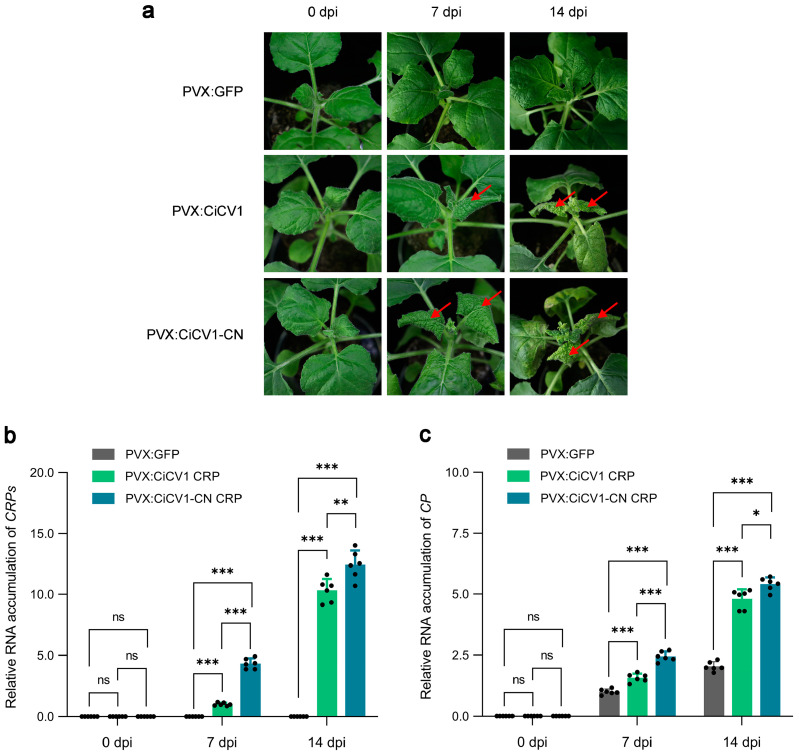
Cysteine-rich protein (CRP) encoded by the Chinese isolate of *Carya illinoinensis* carlavirus 1 (CiCV1-CN) is a pathogenicity factor of CiCV1-CN. (**a**) Roles of CiCV1 CRP and CiCV1-CN CRP in the modulation of symptom development in *Nicotiana benthamiana*. Wild-type *N. benthamiana* seedlings were agro-inoculated with potato virus X (PVX):CiCV1 CRP or PVX:CiCV1-CN CRP at 7 and 14 days post-infiltration (dpi). *N. benthamiana* seedlings agro-inoculated with the PVX-based vector expressing a green fluorescent protein (GFP) were used as negative controls. The red arrows indicate disease symptoms caused by PVX:CiCV1 CRP or PVX:CiCV1-CN CRP infection. (**b**) Quantitative PCR (qPCR) analysis of the RNA accumulation of *CRPs* encoded by CiCV1 and CiCV1-CN in systemic leaves shown in (**a**). (**c**) qPCR analysis of the RNA accumulation of the PVX *coat protein* (*CP*) gene in systemic leaves shown in (**a**). For b and c, *N. benthamiana actin 2* (*NbACT2*) was used as an internal reference. The data are presented as means ± standard deviation of three biological replicates. Significant differences in expression are marked with asterisks: * *p* < 0.01, ** *p* < 0.01, or *** *p* < 0.001; Student′s *t*-test. ns, not significant.

To further determine the roles of CiCV1 CRP and CiCV1-CN CRP in the viral infection, we transiently expressed them in wild-type *N. benthamiana* plants using the PVX-based vector. Compared with *N. benthamiana* seedlings agro-inoculated with PVX:GFP, plants agro-inoculated with either PVX:CiCV1 CRP or PVX:CiCV1-CN CRP showed typical downward leaf curl at 7 dpi, especially those agro-inoculated with PVX:CiCV1-CN CRP (Figure 5a). Notably, *N. benthamiana* seedlings agro-inoculated with both PVX:CiCV1 CRP, and PVX:CiCV1-CN CRP exhibited a severe downward leaf curl with hypersensitive cell death at 14 dpi (Figure 5a). To verify the above observations, we determined the RNA accumulation of *CRPs* of CiCV1 and CiCV1-CN using qPCR. The results showed that *CRP* transcripts were significantly accumulated in the systemic leaves of *N. benthamiana* plants agro-inoculated with either PVX:CiCV1 CRP or PVX:CiCV1-CN CRP at 7 and 14 dpi (Figure 5b). Furthermore, we also found that the transcripts of PVX *CP* were dramatically accumulated in the systemic leaves of *N. benthamiana* plants with prolongation of inoculation time, especially in those of agro-inoculation with PVX:CiCV1 CRP and PVX:CiCV1-CN CRP (Figure 5c), indicating that CiCV1 CRP and CiCV1-CN CRP can increase the viral titer of PVX. Together, these results suggest that the transient expression of CiCV1-CN CRP can induce viral symptoms in *N. benthamiana*, and CiCV1-CN CRP is a pathogenicity factor of CiCV1-CN during viral infection.

## 4. Discussion

Previous studies have shown that the chrysanthemum is one of the most susceptible plants to viral infections, and more than 20 viruses and viroids have been identified to infect the chrysanthemum plants up to now [6,13,16]. In this study, a new full-length isolate of CiCV1 with 8795 nt was identified in *C. morifolium* from Zhejiang Province, China, which was tentatively named the Chinese isolate of CiCV1 (CiCV1-CN). The genome of CiCV1-CN contained a 68-nt 5′-UTR and a 76-nt 3′-UTR, and encoded six putative viral proteins (Figure 1d). According to the species demarcation criteria established for the *Carlavirus* genus [27,54,55], CiCV1-CN is a new carlavirus species with a closer evolutionary relationship with *Carlavirus* CVR (Figure 2 and Figure 3).

Currently, the *Carlavirus* genus comprises 67 species, according to the International Committee on Taxonomy of Viruses (https://ictv.global/, accessed on 16 April 2023) [57]. The genus *Carlavirus* belongs to the subfamily *Quinvirinae*, whose members contain five conserved proteins (a replicase, three TGBps, and a CP) and a variable CRP protein [13,19,24,26,31,54,58,59]. Interestingly, all carlaviruses identified from chrysanthemum plants contain the CRP protein [6,13,19,24,31,33]. In our study, CiCV1-CN has also been demonstrated to possess the CRP protein that shows a higher level of sequence conservation among members of the *Carlavirus* identified from *C. morifolium* (Table 1 and Figure 4).

It has been shown that viral symptoms caused by the genus *Carlavirus* depend mainly on the types of viruses and host plants [26,29,60]. Previous studies have revealed that CRPs encoded by carlaviruses isolated from *C. morifolium* are responsible for symptom generation during viral infections [6,13,33]. For example, the CRP encoded by CVB (CVB CRP) is frequently characterized as a pathogenicity factor and a viral suppressor of RNA silencing, and the transient overexpression of CVB CRP using a PVX-based vector in *N. tabacum* and *N. benthamiana* plants can induce a hypersensitive response [30,56,61,62]. In our study, when CiCV1-CN CRP was transiently expressed in *N. benthamiana* using a PVX-based vector, severe downward leaf curl and hypersensitive cell death were observed in the PVX:CiCV1-CN CRP-inoculated plants (Figure 5). These results provide new evidence that CRPs encoded by carlaviruses isolated from *C. morifolium* are pathogenicity factors. These findings improve our understanding of the molecular mechanisms of the transmission and pathogenesis of carlaviruses.

During the past decade, NGS technology has been extensively utilized to facilitate the discovery of new viruses and viroids [63,64,65,66,67,68]. Many new plant virus species have been identified and characterized using the NGS, including viruses that infect crops, vegetables, ornamentals, and tree plants [69,70,71]. NGS technology has also been used to identify new isolates and variants of existing plant viruses, which have emerged due to the high mutation rates and genetic diversity among plant viruses [64,66,72]. In addition, NGS-based diagnostic and screening tools have been developed to detect these new viruses quickly and accurately, facilitating their management and control in crop production [66,67,68,72]. Collectively, NGS technology has opened up new avenues for the detection, discovery, and characterization of plant viruses. The continued implementation and advancement of NGS technology in plant virology will undoubtedly lead to new discoveries and improved methods for managing plant viral diseases.

## 5. Conclusions

In this study, we reported the complete genome sequence of a Chinese isolate of CiCV1 (CiCV1-CN) from *C. morifolium* plants in Zhejiang Province, China, the main *C. morifolium* production region, with molecular properties to those of members of the *Carlavirus* genus. Furthermore, we confirmed that the CiCV1-CN CRP protein is a pathogenicity factor of CiCV1-CN and can elicit hypersensitive cell death in *N. benthamiana* plants. These results also revealed that the combination of NGS, bioinformatics, and PVX-based expression analysis is a helpful method for discovering new hosts for plant viruses.

## Figures and Tables

**Table 1:** Nucleotide and protein sequence identities (%) of the Chinese isolate of *Carya illinoinensis* carlavirus 1 (CiCV1-CN) and the other 17 carlaviruses from *C. morifolium*.

Viruses	Accession No.	Genome (nt ^a^)	5′-UTR (nt ^a^)	3′-UTR (nt ^a^)	ORF1 (aa ^b^)	ORF2 (aa ^b^)	ORF3 (aa ^b^)	ORF4 (aa ^b^)	ORF5 (aa ^b^)	ORF6 (aa ^b^)
CVR-X6	MZ514906	71.3%	79.2%	84.8%	76.7%	80.3%	71.0%	52.4%	90.2%	71.3%
CVR-X13	MZ514907	71.1%	71.0%	84.8%	77.0%	80.3%	70.1%	52.4%	89.3%	72.2%
CVR-BJ	MG432107	71.0%	84.3%	87.3%	76.8%	78.6%	73.8%	60.9%	88.9%	78.5%
CVR-X21	MZ514905	70.9%	71.0%	84.8%	77.1%	76.9%	74.8%	54.0%	89.3%	79.4%
CVR-ZJHU1	ON137989	70.7%	84.5%	87.3%	76.0%	76.0%	72.9%	54.0%	89.6%	77.6%
CVR-ZJHU2	ON137990	70.4%	84.5%	87.3%	76.2%	75.5%	73.8%	58.7%	89.9%	77.6%
CVR-TX	MN652896	70.4%	84.5%	88.5%	76.2%	76.0%	73.8%	54.0%	90.2%	78.5%
CVB-UK	AM765838	60.9%	NA ^c^	44.6%	61.8%	52.4%	49.5%	25.0%	39.8%	42.5%
CVB-UP	AM765837	60.7%	NA ^c^	44.6%	62.1%	52.4%	49.5%	31.3%	41.9%	37.2%
CVB-PB	AM493895	60.6%	NA ^c^	44.6%	61.7%	51.3%	49.5%	32.8%	46.1%	42.5%
CVB-TN	AM765839	60.5%	NA ^c^	44.6%	61.6%	53.9%	49.5%	23.5%	43.6%	42.5%
CVB-FY	MZ514910	56.9%	47.1%	67.9%	47.0%	53.7%	50.5%	29.9%	46.7%	42.5%
CVB-GS2	MZ514909	56.4%	50.7%	69.0%	47.0%	52.8%	50.5%	26.9%	45.2%	38.8%
CVB-GS1	MZ514908	56.4%	50.7%	69.0%	47.3%	53.9%	49.5%	20.6%	45.8%	42.5%
CVB-CN5	MW691877	56.3%	60.0%	67.9%	47.1%	54.7%	49.5%	22.1%	41.9%	38.3%
CVB-S	AB245142	56.3%	60.0%	70.2%	46.7%	52.6%	49.5%	26.9%	46.4%	40.5%
CVB-CN2	MW691876	56.2%	60.0%	67.9%	47.1%	54.3%	49.5%	22.1%	44.7%	39.2%

^a^ nt, nucleotide; ^b^ aa, amino acid; ^c^ NA, not applicable.

## Data Availability

The genome sequence of the Chinese isolate of *Carya illinoinensis* carlavirus 1 (CiCV1-CN) has been deposited in the NCBI GenBank (https://www.ncbi.nlm.nih.gov/genbank/ (accessed on 7 February 2023)) under an accession number OQ410649. The other data presented in this study are available in this manuscript.

## Supplementary Materials

The following supporting information can be downloaded at: https://www.mdpi.com/article/10.3390/v15041029/s1, Figure S1: Alignments of amino acid sequences coat proteins (CPs) of the Chinese isolate of *Carya illinoinensis* carlavirus 1 (CiCV1-CN) and the other eight carlaviruses; Table S1: List of primers used in this study; Table S2: Summary of the next-generation sequencing (NGS) of *Chrysanthemum morifolium* leaves infected with the Chinese isolate of *Carya illinoinensis* carlavirus 1 (CiCV1-CN); Table S3: Contigs mapped in the genome of the Chinese isolate of *Carya illinoinensis* carlavirus 1 (CiCV1-CN); Table S4: Nucleotide and protein sequence identities (%) of the Chinese isolate of *Carya illinoinensis* carlavirus 1 (CiCV1-CN) and 31 reported carlaviruses; Table S5: Recombination analysis of the Chinese isolate of *Carya illinoinensis* carlavirus 1 (CiCV1-CN) and 31 reported carlaviruses using the RDP4 software.
