# Supplementary material for: Identification and Molecular Characterization of a Novel Carlavirus Infecting Chrysanthemum morifolium in China
Source: Viruses. 2023 Apr 21;15(4):1029. doi: 10.3390/v15041029 (PMC10141686; doi:10.3390/v15041029)
Supplement: Supplementary file 1 [file viruses-15-01029-s001.zip › Supplementary Figure S1.pdf]

Supplementary Figure S1

a

	CiCV1-CN CP	CiCV1 CP	CVR-ZJHU1 CP	CVR-ZJHU2 CP	CVR-TX CP	CVR-BJ CP	CVR-X21 CP	CVR-X6 CP	CVR-X13 CP
CiCV1-CN CP	–	96.4%	89.6%	89.9%	90.2%	88.9%	89.3%	90.2%	89.3%
CiCV1 CP	96.4%	–	88.6%	88.9%	89.3%	88.3%	88.9%	89.3%	88.3%
CVR-ZJHU1 CP	89.6%	88.6%	–	99.3%	99.3%	94.5%	94.8%	91.9%	90.6%
CVR-ZJHU2 CP	89.9%	88.9%	99.3%	–	99.3%	94.5%	94.8%	92.2%	90.9%
CVR-TX CP	90.2%	89.3%	99.3%	99.3%	–	95.1%	95.4%	92.5%	91.2%
CVR-BJ CP	88.9%	88.3%	94.5%	94.5%	95.1%	–	98.0%	92.8%	91.9%
CVR-X21 CP	89.3%	88.9%	94.8%	94.8%	95.4%	98.0%	–	93.5%	92.5%
CVR-X6 CP	90.2%	89.3%	91.9%	92.2%	92.5%	92.8%	93.5%	–	98.7%
CVR-X13 CP	89.3%	88.3%	90.6%	90.9%	91.2%	91.9%	92.5%	98.7%	–

b

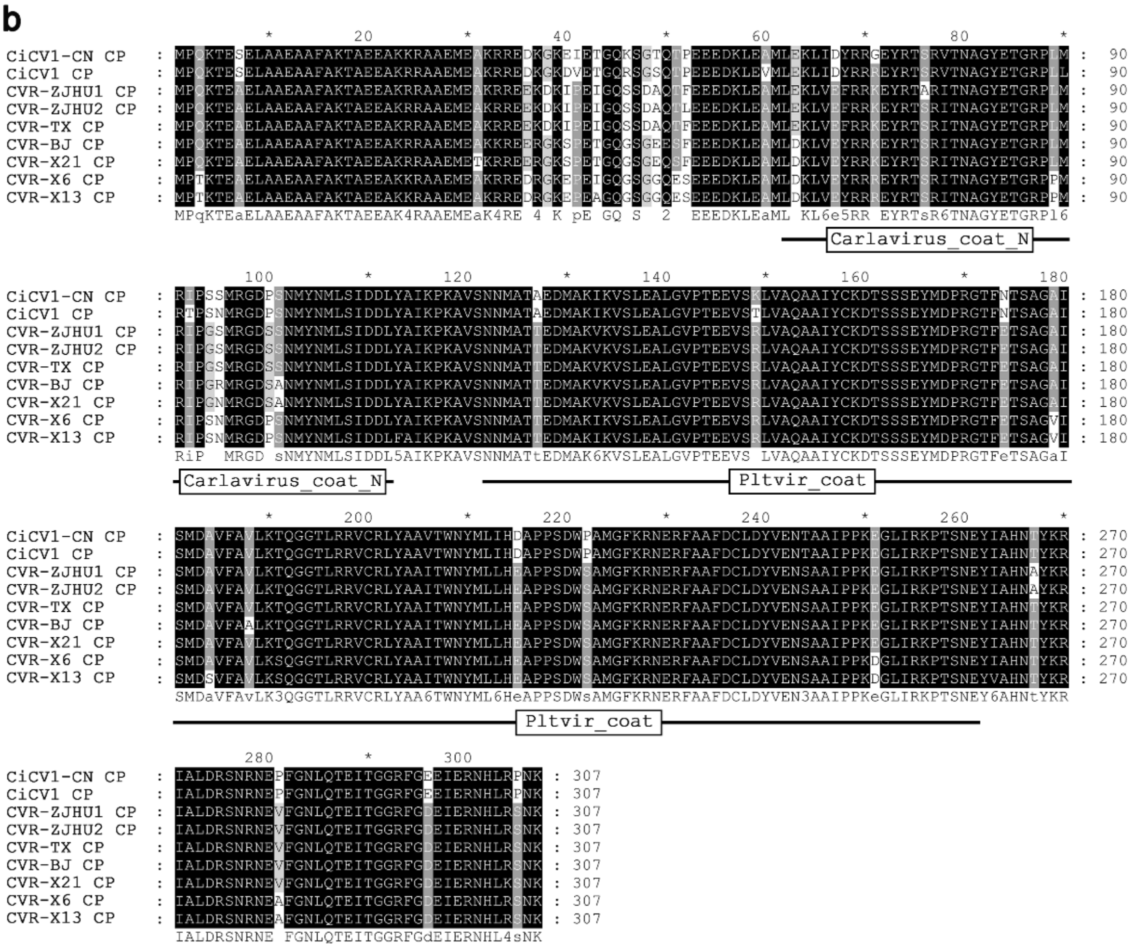

Figure S1. Alignments of amino acid sequences coat proteins (CPs) of the Chinese isolate of *Carya illinoinensis* carlavirus 1 (CiCV1-CN) and the other eight carlaviruses. (**a**) Pairwise sequence alignment of the CPs of the carlaviruses using the EMBOSS Needle (https://www.ebi.ac.uk/Tools/psa/emboss_needle/). (**b**) Amino acid sequence alignment of the CPs of the carlaviruses using the ClustalW program embedded in Molecular Evolutionary Genetics Analysis software (MEGA, v11.0), and the conserved domains were determined using the InterPro (http://www.ebi.ac.uk/interpro/). The following viruses were used in the amino acid sequence alignments: *Carya illinoinensis* carlavirus 1 (CiCV1, MW328759), *Carya illinoinensis* carlavirus 1 Chinese isolate (CiCV1-CN, OQ410649), chrysanthemum virus R isolate TX (CVR-TX, MN652896), chrysanthemum virus R isolate X13 (CVR-X13, MZ514907), chrysanthemum virus R isolate X21 (CVR-X21, MZ514905), chrysanthemum virus R isolate X6 (CVR-X6, MZ514906), chrysanthemum virus R isolate ZJHU1 (CVR-ZJHU1, ON137989), chrysanthemum virus R isolate ZJHU2 (CVR-ZJHU2, ON137990), and chrysanthemum virus R isolate BJ (CVR-BJ, MG432107).
